# Adherence to a Fish-Rich Dietary Pattern Is Associated with Chronic Hepatitis C Patients Showing Low Viral Load: Implications for Nutritional Management

**DOI:** 10.3390/nu13103337

**Published:** 2021-09-23

**Authors:** Claudia Ojeda-Granados, Arturo Panduro, Karina Gonzalez-Aldaco, Ingrid Rivera-Iñiguez, Liliana Campos-Medina, Sonia Roman

**Affiliations:** 1 Department of Molecular Biology in Medicine, Civil Hospital of Guadalajara “Fray Antonio Alcalde”, Hospital #278, Col. El Retiro, Guadalajara 44280, Jalisco, Mexico; claudiaojedagranados@hotmail.com (C.O.-G.); apanduro@prodigy.net.mx (A.P.); karinaldaco@hotmail.com (K.G.-A.); ingrid_rivei@hotmail.com (I.R.-I.); lilianacamposm@gmail.com (L.C.-M.); 2 Health Sciences Center, University of Guadalajara, Guadalajara 44340, Jalisco, Mexico

**Keywords:** hepatitis C virus, lipid metabolism, dietary pattern, nutritional profile, APOE, polyunsaturated fatty acid, fiber, spontaneous clearance, fibrosis

## Abstract

Hepatitis C virus (HCV) infection is influenced by genetic (e.g., *APOE* polymorphisms) and environmental factors between the virus and the host. HCV modulates the host’s lipid metabolism but dietary components influence lipids and in vitro HCV RNA replication. Few data exist on the role of dietary features or patterns (DPs) in HCV infection. Herein, we aimed to evaluate the nutritional profiles of chronic HCV (CHC) and spontaneous clearance (SC) Mexican patients in the context of *APOE* alleles and their correlation with HCV-related variables. The fibrosis-related *APOE* *ε3* allele prevailed in CHC and SC patients, who had four DPs (“meat and soft drinks”, DP1; “processed animal and fried foods”, DP2; “Mexican-healthy”, DP3; and “fish-rich”, DP4). In CHC subjects, polyunsaturated fatty acid intake (PUFA ≥ 4.9%) was negatively associated, and fiber intake (≥21.5 g/day) was positively associated with a high viral load (*p* < 0.036). High adherence to fish-rich DP4 was associated with a higher frequency of CHC individuals consuming PUFA ≥ 4.9% (*p* = 0.004) and low viral load (*p* = 0.036), but a lower frequency of CHC individuals consuming fiber ≥21.5 g/day (*p* = 0.038). In SC and CHC individuals, modifying unhealthy DPs and targeting HCV-interacting nutrients, respectively, could be part of a nutritional management strategy to prevent further liver damage.

## 1. Introduction

Chronic hepatitis C virus (HCV) infection is a leading cause of chronic liver disease along with hepatitis B virus infection, alcohol-related liver disease, and the emerging metabolic-associated fatty liver disease [1,2,3]. According to the World Health Organization, 58 million people are chronically infected, and 290,000 deaths per year are related to HCV infection [1]. Chronic HCV (CHC) infection is epidemiologically heterogenous in regard to prevalence rates and associated risk factors. Furthermore, interactions between the distinct virus types (genotypes 1–7) and their putative host populations are regulated by genetic, immune, and metabolic factors influencing the natural course of HCV infection [4]. In this sense, there is increasing evidence that HCV modulates the host’s lipid metabolism to enhance its life cycle [5]. HCV favors a lipid-rich intrahepatocyte but lipid-depleted extrahepatic environment by promoting lipogenesis, down-regulating fatty acid oxidation, and impairing very-low-density lipoprotein (VLDL-c) secretion [6,7]. The resulting metabolic phenotype in chronically infected patients is often characterized by serum hypocholesterolemia, hypobetalipoproteinemia, and in some cases, liver steatosis, insulin resistance, or type 2 diabetes mellitus [8,9].

As a complex process, lipid metabolism is in turn influenced by environmental factors, such as diet [10]. For example, Western-style diets, typically high in energy intake, saturated fatty acids (SFA), cholesterol, simple sugars, and an increased ratio of n-6:n-3 polyunsaturated fatty acids (PUFA) have been shown to increase the risk of chronic inflammatory non-communicable diseases such as obesity, T2DM, and cardiovascular diseases, metabolic-associated fatty liver disease, as well as certain cancers [11,12]. Notably, some specific dietary components enhance in vitro HCV RNA replication, such as excess SFA (e.g., lauric, myristic, and palmitic) or monounsaturated fatty acids (MUFA), vitamin E, and resveratrol [13,14]. Conversely, some foods or nutrients, denoted as “anti-HCV nutrients” (i.e., PUFA-rich fish, n-3 PUFA supplementation, linoleic, arachidonic, docosahexaenoic, and eicosapentaenoic PUFA, β-carotene, vitamin D, and gallic acid) have been shown to interfere with viral replication mechanisms [13,15,16,17,18] or to have protective effects against adverse health outcomes associated with HCV infection [19,20]. Additionally, some key genetic and metabolic factors associated with HCV spontaneous clearance (SC) status or less liver damage in chronic HCV (CHC) patients [21,22] have emerged. Hypercholesterolemia associated with the apolipoprotein E (*APOE*) *ε4* allele, and overweight have been reported to be more prevalent in SC subjects [23]. Likewise, the *ε4* allele in CHC has been related to hypercholesterolemia but less liver damage than the *ε3* allele, carriers of which show advanced fibrosis [23].

Diverse nations around the world, including Mexico, have shifted their regional food culture towards a globalized Western-style diet or pattern [24,25,26,27]. In Mexico, it is denoted as a hepatopathogenic diet due to its association with the onset and progression of liver damage in non-HCV patients [27,28]. Indeed, Western-like diets or patterns have been associated with a worse metabolic profile, including increased insulin resistance, blood pressure, and liver fibrosis, in HCV patients, indirectly impairing the response to antiviral treatment, as well as resulting in a direct risk of hepatocellular carcinoma [29,30,31]. Nonetheless, no data exist on dietary features or dietary patterns (DPs) in anti-HCV-positive patients and the likely impact on their clinical profile when coupled with a lipid-related genetic factor such as *APOE*. In this study, we aimed to analyze the nutritional profiles of HCV treatment-naive Mexican patients in the context of *APOE ε2*, *-ε3*, and *-ε4* alleles and to assess their correlation with the clinical outcomes of HCV infection.

## 2. Materials and Methods

### 2.1. Study Design and Patients

A detailed description of the study protocol complying with the Declaration of Helsinki ethical guidelines and approved by the Institutional Review Board (Health Sciences Center, University of Guadalajara, Certificate #CI-00612) is provided elsewhere [23]. Briefly, from January 2014 to December 2016, adult patients were included in the study if they were serologically positive for anti-HCV antibodies, treatment-naive, and unrelated. The exclusion criteria were chronic hepatitis B virus or human immunodeficiency virus infections, autoimmune liver disease, Child–Pugh classes B and C, Wilson’s disease, hemochromatosis, drinkers, and use of hypolipidemic drugs. Patients were enrolled at the Nutrigenetic Clinic, Department of Molecular Biology in Medicine, Civil Hospital of Guadalajara “Fray Antonio Alcalde” in Guadalajara, Mexico. After passing exclusion criteria and undergoing quantitative serum HCV RNA assessment by RT-qPCR (Roche COBAS^®^ AmpliPrep and COBAS^®^ TaqMan 48 HCV test, Pleasanton, CA, USA), individuals were grouped as SC or CHC patients if they had at least two undetectable serum HCV RNA results or if they had two detectable serum HCV RNA results, respectively, in the preceding 12 months with a six-month interval between tests.

For this secondary retrospective analysis, anti-HCV-positive patients from the first cohort (SC and CHC) with data on demographic, clinical, and genetic features (i.e., genotyping of *APOE* rs429358 and rs7412 polymorphisms), as well as their nutritional assessment, were included to analyze their dietary profiles and their clinical correlations in the context of the *APOE*-*ε2*, -*ε3*, and -*ε4* alleles.

### 2.2. Clinical Data

Clinical data comprised the anthropometric and biochemical features of patients. Anthropometric variables included height, measured with a stable stadiometer (seca GmbH & Co. KG, Hamburg, Germany), and body composition parameters (i.e., weight, body fat percentage, and body mass index, BMI) that were determined by bioelectrical impedance analysis using the InBody 3.0 analyzer (InBody Co., Seoul, Korea). Normal weight (≥18.5–24.99 kg/m^2^), overweight (≥25.0–29.99 kg/m^2^), and obesity (≥30.0 kg/m^2^) were defined according to BMI and World Health Organization criteria.

Laboratory tests were performed on venous blood samples collected after a 12 h overnight fast to determine platelet count, total bilirubin, and total cholesterol, and concentrations of glucose, albumin, triglycerides, high-density lipoprotein cholesterol (HDL-c), alanine aminotransferase (ALT), aspartate aminotransferase (AST), and gamma-glutamyl transferase (GGT) through a biochemical analyzer (Vitros 250, Ortho Clinical Diagnostics, Johnson & Johnson, Rochester, NY, USA). The LDL-c level was estimated with the Friedewald formula [32], and VLDL-c as total cholesterol (LDL-c + HDL-c). The biochemical variables of glucose, platelet count, albumin, total bilirubin, and GGT previously associated with liver damage [33] were considered in CHC subjects.

### 2.3. Lipid Profile Abnormalities

Hypercholesterolemia (≥200 mg/dL), hypertriglyceridemia (≥150 mg/dL), elevated LDL-c (≥130 mg/dL), and hypoalphalipoproteinemia (HDL-c ≤ 40 mg/dL in men and ≤50 mg/dL in women) were diagnosed by considering the criteria of the National Cholesterol Education Program ATP III [34] and the Norma Oficial Mexicana NOM-037-SSA2-2012 for the treatment of dyslipidemia [35].

### 2.4. APOE Genotyping

The *APOE* rs429358 and rs7412 polymorphisms leading to the common *ε2*, *ε3*, and *ε4* alleles were determined by allelic discrimination assays and real-time PCR using genomic DNA samples isolated from white blood cells. In particular, the predesigned TaqMan^®^ SNP Genotyping Assays C___3084793_20 and C____904973_10 (Applied Biosystems, Life Technologies, Foster City, CA, USA) were employed to perform the experiments on a StepOnePlus thermocycler (Applied Biosystems, Life Technologies, Foster City, CA, USA) by setting an initial enzyme activation step for 10 min at 95 °C, followed by 40 cycles of denaturalization for 15 s at 95 °C and annealing/extension for 1 min at 60 °C. The *APOE ε2*, -*ε3*, and -*ε4* allele frequencies were derived by direct counting. For analysis purposes and according to the known contribution of *APOE* alleles to the lipid profile, *APOE* genotypes were classified as E2 (*ε2ε2* + *ε2ε3* + *ε2ε4*), E3 (*ε3ε3*), or E4 (*ε3ε4* + *ε4ε4*) genotype groups.

### 2.5. Nutritional Assessment

Two dietary assessment tools were administered to evaluate the patients’ dietary profile, the 24 h recall to determine their habitual intake, and the food frequency questionnaire (FFQ) to derive their dietary patterns through a data-driven method (i.e., exploratory factor analysis, EFA) [36].

In detail, three 24 h recalls (two weekdays and one weekend day) were administered to participants by trained dietitians as an in-person interview [37]. Dietary recall methodology consisted of five steps: (1) the quick list—subjects were asked to recall all foods and beverages consumed during the previous 24 h; (2) the forgotten foods list—subjects were asked to recall on categories of foods that have been documented as frequently forgotten; (3) time/occasion—subjects were asked to recall the time and occasion at which foods were consumed; (4) the detail cycle—participants were asked to give details related to the amount, portion sizes, characteristics, preparation styles and recipes of the foods and beverages consumed; and (5) the final probe—probes of portion sizes of foods and beverages using measuring cups to have a better estimation.

Collected data from the 24 h recalls were then processed with NutriKal^®^ VO software (Consinfo S.C., CDMX, Mexico) to estimate patients’ daily energy and macro- and micronutrient intake for correlation analyses. This software uses foods from the USDA National Nutrient Database for Standard Reference, Release 24 [38] and contains the composition tables compiled by the Instituto Nacional de Ciencias Médicas y Nutrición Salvador Zubirán (INCMNSZ) and the information derived from the Mexican Equivalent Food System [39], as well as the information from industrialized foods available in our country and the result of the nutritional calculation of typically consumed dishes.

### 2.6. Dietary Pattern Analysis

Dietary information from a 64-item adjusted FFQ [40], with a five-choice frequency scale comprising daily, 1–2 times per week, ≥3 times per week, once every 15 days, and once per month, was instead considered to derive typical DPs among anti-HCV-positive patients. The 64 FFQ items were reorganized into 23 food groups based on the similarity of their nutritional content. DPs were generated using the EFA method [36]. To this end, the adequacy of the sample size was first checked using the Kaiser–Meyer–Olklin (KMO) test, considering a value of ≥0.60 as acceptable [41]. Then, a principal axis factor extraction method and an orthogonal varimax factor rotation strategy were applied for the EFA [42]. A parallel analysis was conducted to determine the number of factors (i.e., dietary patterns) to be selected [41]. Accordingly, only factors (dietary patterns) with original eigenvalues above their corresponding randomly generated eigenvalues (95th percentile value) were retained [43]. Next, to identify variables (i.e., food groups) as highly representative of the identified DPs, a cutoff value of ≥0.30 for factor loadings was considered indicative of a strong association between a food group and DP [44]. Thus, DPs were named considering the most representative food groups in it. Finally, to analyze the relationship between adherence to the identified DPs and variables associated with HCV infection, DP factor scores obtained for each individual and representing the level of adherence to the identified DPs were classified into tertiles (T1, T2, and T3), with values in the T3 tertiles being associated with greater adherence.

### 2.7. Statistical Analysis

Statistical analyses and EFA were conducted using the IBM SPSS v.20 software (IBM Corp, Inc., Chicago, IL, USA). A *p*-value < 0.05 was considered for statistical significance on two-sided tests. Categorical variables were expressed as frequencies and percentages and analyzed with chi-square test, while continuous variables were analyzed as means ± standard deviation. The normality distribution of quantitative variables was assessed with the Kolmogorov–Smirnov test with Lilliefors significance correction. Thus, variables with normal and non-normal distributed data were analyzed with Student’s *t*-test and Mann–Whitney U test, respectively. For the latter, the median, first quartile (Q1), and third quartile (Q3) were also reported. Differences in nutritional profile variables according to the *APOE* genotype group in CHC and SC patients were evaluated with the non-parametric Kruskal–Wallis test, because there were some genotype groups with fewer than 30 patients. A value <600,000 IU/mL of HCV RNA, referred to as low baseline viral load [45], was considered to classify CHC patients into low or high (≥600,000 IU/mL) viral load.

The dietary variables associated with HCV viral load were identified by univariate and multivariate logistic regression analysis. Results were expressed as an odds ratio with a 95% confidence interval. The robustness of the regression model was tested using the Hosmer–Lemeshow method. An area under the receiver-operating characteristic (ROC) curve analysis was performed to determine optimal cutoff values for variables associated with HCV viral load. Differences in the frequency of CHC patients with HCV-related variables concerning adherence to identified DPs were assessed with a chi-square test.

## 3. Results

### 3.1. Demographic, Clinical, and Genetic Characteristics of Patients

The clinical and genetic characteristics of anti-HCV-positive patients are depicted in Table 1. No significant differences in gender, body fat percentage, or BMI were found between CHC and SC groups. However, according to BMI classification, CHC patients were more frequently in the normal weight range than SC subjects (34.8% vs. 19.6%, *p* = 0.045). Consistent with the peculiar lipid and metabolic profile related to chronic infection, the CHC group showed lower levels of total cholesterol, triglycerides, LDL-c, and VLDL-c, but increased ALT, AST, and GGT compared to the SC group (*p* < 0.001). In contrast, lipid abnormalities (i.e., hypercholesterolemia, hypertriglyceridemia, and elevated LDL-c) were more frequent in SC than in CHC patients (*p* < 0.020).

Regarding the *APOE* genotyping data, no differences were observed in the distribution of genotype or allele frequencies between the CHC and SC groups. *APOE ε3* allele was the most frequent among both groups, whereas the frequency of the *ε4* allele was higher in SC subjects than in CHC subjects.

### 3.2. Nutritional Profile of CHC and SC Patients According to APOE Genotype Group

The nutritional profile, including anthropometric and dietary features, of CHC and SC patients according to their *APOE* genotype group is displayed in Table 2. No significant differences were observed in the nutritional profile of CHC or SC subjects according to their *APOE* genotype group. However, both groups of patients showed dietary features typical of a hepatopathogenic diet, such as a high mean intake of SFA, cholesterol, carbohydrates, and added sugars, and a low mean intake of MUFA, PUFA, and fiber. Compared to the Recommended Dietary Allowance (RDA) values [46], the mean intake of some micronutrients with a potential effect on liver function, particularly vitamin E, folates, pyridoxine, and selenium, was deficient.

### 3.3. Identification of Dietary Patterns among CHC and SC Patients

**Figure 1:**
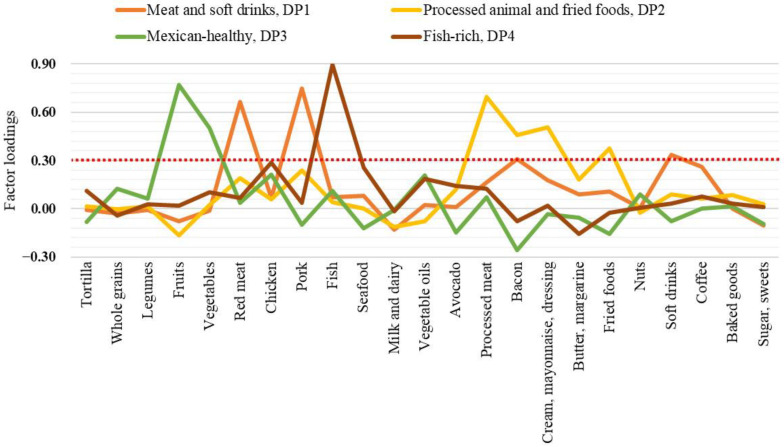
Dietary patterns in anti-HCV-positive patients identified by exploratory factor analysis. The red dotted line indicates the cutoff value of the factor loadings in which a food group was considered highly representative of the dietary pattern.

To identify the food groups that contribute the most to the habitual dietary characteristics of CHC and SC subjects, an EFA was conducted on data from a 64-item FFQ, reorganized into 23 food groups. A KMO test value of 0.60 was obtained, indicating an acceptable sample size to run the EFA. According to the parallel post-EFA analysis, four main factors (i.e., dietary patterns) were identified, which accounted for 36.9% of the total variance. Each DP was named considering the kind of those food groups representative in it based on the factor loading scores cutoff value of ≥0.30 (Figure 1 and Supplementary Table S1).

Accordingly, the meat and soft drinks DP1 was mainly characterized by pork, red meat, soft drinks, and animal fat (bacon) and the processed animal and fried foods DP2 featured processed meat, animal fats (cream, mayonnaise, dressings, and bacon), and fried foods. These DPs, primarily represented by Western-type foods, accounted for 13.8% and 8.2% of the total variance, respectively. The Mexican-healthy DP3 was characterized mainly by fruits and vegetables, although chicken, vegetable oils, and whole grains were also present. Finally, the fish-rich DP4 was strongly defined by fish, but seafood and vegetable oils were also found. The later accounted for 7.6% and 7.3% of the total variance.

### 3.4. Association of Nutritional and Biochemical Characteristics with the Clinical Outcome of HCV Infection

The relationship between some nutritional profile characteristics and biochemical variables, previously related to liver damage, with low or high viral load in CHC patients, was evaluated (Table 3). CHC subjects with low viral load had significantly higher intakes of total fat and PUFA, while those with high viral load had a higher fiber intake and showed higher ALT and GGT.

Univariate and multivariate logistic regression analysis was then performed to assess the association of the most relevant dietary characteristics with a high viral load (Table 4). PUFA intake was negatively associated, and fiber intake was positively associated with a high viral load (*p* < 0.036). The area under the ROC curve analysis was conducted to estimate the optimal intake cutoff values of PUFA and fiber related to a low and high viral load, respectively (Table 5).

### 3.5. Association of Adherence to Dietary Patterns with HCV-Related Variables

Finally, considering the estimated cutoff values of PUFA and fiber that were found to be associated with HCV viral load, the relationship between adherence to the identified dietary patterns and the frequency of patients with low or high viral load as well as PUFA and fiber intake corresponding to the cutoff value was evaluated (Table 6). Only high adherence (T3) to the fish-rich DP4 was associated with a higher frequency of patients with PUFA intake ≥4.9% and low viral load, and in turn, a lower frequency of patients with fiber intake ≥21.5 g/day compared to low adherence (T1) to such dietary pattern.

## 4. Discussion

The clinical outcomes of HCV infection are modulated by the interplay of genetic and environmental factors between the virus and the host. On the one hand, HCV is genetically classified into seven genotypes and >40 subtypes with a heterogeneous geographic distribution (different hosts’ ethnicities). Such genetic variability has pathogenic and clinical implications [47]. On the other hand, several human genes are known to modulate the immune response system, lipid transport, and lipid metabolism, as well as liver fibrogenesis driven by HCV [4,8,48]. Ultimately, diet composition is an environmental factor that contributes to the onset and progression of liver disease in susceptible individuals and has been less explored in vivo in the context of CHC infection [29,30,49]. Studies in vitro have shown that HCV RNA replication can be modulated by the quality and quantity of certain macro- and micronutrients, some of which can directly or indirectly influence lipid metabolism [13,14,50].

This study revealed that, regardless of *APOE* allele type, both CHC and SC patients consumed a hepatopathogenic-type diet, in line with earlier findings from studies conducted in non-HCV patients with obesity-related dysfunctional metabolism [28,51]. This diet was rich in SFA, cholesterol, carbohydrates, and added sugars but deficient in MUFA, PUFA, fiber, and micronutrients with likely beneficial effects on liver function, evidencing that despite their medical condition, this group of patients is equally immersed in the obesogenic environment surrounding the Mexican population [52]. Furthermore, the paradoxically protective *APOE ε4* allele in anti-HCV-positive subjects showed the frequency typical of the admixed population of West Mexico [53], but consistent with earlier findings [23], it was higher in SC patients, who also showed a higher frequency of lipid profile abnormalities than CHC subjects. However, the fibrosis-related *APOE ε3* allele was the most frequent among both groups. Thus, besides the potentially risky *APOE ε3* genetic factor prevalent in this population group, a hepatopathogenic-type diet might contribute to an increased risk of liver damage progression.

The identification of dietary patterns has been considered a more realistic representation of the food type and dietary habits of a population than the isolated consumption of nutrients [54]. Dietary intake follows a pattern of consumption, and the diet itself is a modifiable risk factor. In particular, four dietary patterns (i.e., meat and soft drinks, DP1, processed animal and fried foods, DP2, Mexican-healthy, DP3, and fish-rich, DP4) were identified in CHC and SC patients. DP1 and DP2, characterized mainly by pork, red meat, soft drinks, animal fat (e.g., bacon, cream, mayonnaise, and dressings), processed meat, and fried foods, accounted for the largest variance of dietary patterns. Interestingly, despite the consumption of these Western-like DPs, CHC individuals exhibited the peculiar, lowered profile of cholesterol, triglycerides, LDL-c, and VLDL-c compared to that of SC patients that have been reported by our research group [23] and other authors as well [9,55]. This alteration is explained by the fact that HCV hijacks the lipoprotein pathways exerting a plasmatic lipid-lowering effect even if the patients consume a lipid-rich diet associated with dyslipidemia, but at the cost of exhibiting fatty hepatocytes.

In addition, this study found differences in some dietary characteristics of CHC patients in relation to their viral load. Specifically, subjects with low viral load showed a significantly higher total fat intake than those with high viral load. Moreover, PUFA intake was negatively associated, whereas fiber intake was positively associated with elevated HCV RNA viral load. In this regard, in vitro studies have shown that long-chain PUFA, such as arachidonic acid (20:4 n-6), docosahexaenoic acid (22:6 n-3), and eicosapentaenoic acid (20:5 n-3), suppressed the expression of genes that regulate enzymes involved in hepatic fatty acid and triglyceride biosynthesis, particularly sterol regulatory element-binding protein-1c (SREBP-1c) and fatty-acid synthase, as well as HCV RNA replication [13,56]. These effects were independent of PUFA’s ability to antagonize liver X nuclear receptors (LXR) that transcriptionally regulate SREBPs (i.e., the LXR-SREBP-1c pathway); yet the mechanism of these effects remains unclear [13].

Another in vitro study provided evidence that eicosapentaenoic acid (20:5 n-3) and arachidonic acid (20:4 n-6) counteracted the fatty acid enzyme delta-9 desaturase enhanced effect by which HCV core protein induces steatosis [50]. Although the specific mechanisms underlying the anti-HCV activity of PUFA have not been fully elucidated, it has been suggested that they may be closely related to the downregulatory activity of some hepatic lipogenic genes, which are also key to HCV-induced lipogenesis for its efficient replication cycle [13]. Furthermore, the few and small clinical studies performed so far have yielded inconsistent results. While some have failed to demonstrate the benefits of PUFA [57,58], others have evidenced the protective effects of n-3 PUFA-rich fish or n-3 PUFA supplementation against detrimental variables associated with HCV infection [19,20].

Concerning fiber consumption, to our knowledge, there are no studies showing its effects in the context of HCV infection. However, we could hypothesize that the association of a fiber intake ≥21.5 g/day with a high viral load might be a collateral effect related to its blood-lipid-lowering properties. Dietary fiber is classified into insoluble and soluble fibers. Insoluble fibers (i.e., lignin, cellulose, and hemicellulose), present in cereals, whole-grain foods, bran, and nuts, help to increase fecal mass, decrease intestinal transit time and serve as a laxative agent when sufficient liquid is consumed [59]. Instead, soluble fibers (e.g., pectin, celluloses, water-soluble gums, fructans, and some resistant starches) present in certain fruits, vegetables, seeds, avocado, oats, and legumes, due to their viscosity and fermentable properties, can reduce postprandial plasma glucose and cholesterol levels through the production of short-chain fatty acids [59]. A fiber-rich diet (>24 g per 1000 kcal), mainly of the soluble type, has been shown to reduce total blood cholesterol and LDL-c levels to their lowest levels, regardless of the amount of other blood lipid dietary modifiers, such as SFA and cholesterol [60]. Therefore, a soluble-type fiber-rich diet might be counterproductive in CHC patients because it may contribute to the lipid-depleted extrahepatic environment that HCV itself promotes.

Treatment of HCV infection has advanced notably in the last decade in terms of developing more effective therapies such as the direct-acting antiviral agents (DAAs) for the promising eradication of HCV while reducing the risk factors involved in liver damage, including necroinflammation, steatosis, cirrhosis, and hepatocellular carcinoma [61]. However, patients living in low-income and middle-income countries such as Latin America do not have immediate access to DDA therapy [62]. Therefore, lifestyle modifications, including diet, are validated resources to manage patients with liver disease [27,30]. Based on the preliminary evidence found in this study, specific nutrients or DPs might play an essential role in modulating the natural course of the infection after the acquisition of HCV, i.e., within the acute phase (first six months) while waiting for confirmation of infection status (chronicity) and during therapy.

In addition, it has recently been documented that patients who achieve a sustained virological response, either by interferon or non-interferon regimens (DAAs), are still at risk of post-therapy clinical complications [63,64]. Furthermore, in the development of chronic liver disease, co-morbidities other than HCV infection, such as obesity, diabetes, metabolic-associated fatty liver, and even co-infection with hepatitis B or HIV, can overlap, acting synergically towards liver damage (cirrhosis) in a single patient. Therefore, the results obtained in this study have implications for the nutritional management of SC and CHC patients focused on improving liver function.

The limitations of the present study were that the specific type of PUFA, fiber, or n-6:n-3 PUFA ratio was not evaluated, nor could we break down all nutrients, specifically vitamin D and iodine related to fish consumption. There was also no possibility to calculate the intake. Nonetheless, despite the retrospective, cross-sectional nature of the study, it revealed that high adherence to the identified fish-rich DP4 was associated with a higher frequency of CHC subjects with PUFA intake ≥4.9% and low viral load, but a lower frequency of individuals consuming ≥21.5 g/day of fiber. Although this DP likely has nutritional characteristics that might benefit this group of patients, further prospective nutrigenetic studies are warranted.

Understanding the wide range of healthy and detrimental dietary patterns worldwide provides valuable knowledge to prevent chronic diseases [65,66,67,68]. However, when designing nutritional interventions strategies containing healthy dietary components, these should be tailored to the population’s genetic background, regional foods, and culinary culture [27,69]. Therefore, in summary, for SC patients with a genetic (*ε4* allele) and metabolic profile related to a higher frequency of lipid abnormalities (i.e., hypercholesterolemia, hypertriglyceridemia, and elevated LDL-c), it is essential to address the hepatopathogenic and Western-like dietary characteristics to prevent further metabolic abnormalities or liver fibrosis progression linked to this type of proinflammatory diet. In CHC patients, in whom the fibrosis-related *ε3* allele is also frequent, in addition to addressing the above, it seems that a lipid-rich diet might also be of benefit, especially one containing long-chain PUFA with an adequate n-6:n-3 ratio, and a fiber intake, mainly of the insoluble type, kept at the lower limit of the daily recommendation. Further large-scale clinical trials are required to confirm these observations or add new evidence.

## Figures and Tables

**Table 1:** Demographics, anthropometrics, lipid profile, and *APOE* characteristics of anti-HCV-positive patients.

	Anti-HCV-Positive Patients			
Variable	Total Study Group (n = 188)	Chronic Hepatitis C (n = 137)	Spontaneous Clearance (n = 51)	*p*-Value
Demographics/Anthropometrics				
Age (years)	49.51 ± 12.0	50.9 ± 11.7	45.7 ± 12.1	0.007
Female/Male n (%)	106/82 (56.4/43.6)	78/59 (56.9/43.1)	28/23 (54.9/45.1)	0.803
Weight (kg)	71.4 ± 14.2	70.4 ± 14.7	74.1 ± 12.5	0.120
Height (cm)	161.5 ± 10.1	161.3 ± 10.3	162.0 ± 9.6	0.689
Body fat mass (kg)	22.3 ± 9.7	21.9 ± 10.2	23.2 ± 8.4	0.417
Percent body fat (%)	30.8 ± 9.9	12.1 ± 2.7	12.7 ± 2.6	0.543
BMI (kg/m^2^)	27.4 ± 5.2	30.5 ± 10.2	31.5 ± 9.1	0.187
Lipid Profile and Liver Enzymes				
Total cholesterol (mg/dL) Median (Q1, Q3)	155.0 ± 49.1 153 (124.0, 183.3)	142.6 ± 45.1 140.0 (116.0, 166.5)	191.0 ± 42. 192.0 (157.0, 214.0)	<0.001 ^a^
Triglycerides (mg/dL) Median (Q1, Q3)	139.6 ± 68.5 123.5 (90.0, 181.0)	128.8 ± 61.1 113.0 (84.0, 166.5)	171.2 ± 79.1 153.0 (108.0, 210.0)	<0.001 ^a^
LDL-c (mg/dL) Median (Q1, Q3)	93.2 ± 37.8 89.5 (67.0, 111.6)	84.8 ± 34.7 82.0 (61.5, 100.0)	118.7 ± 36.1 112.0 (91.0, 136.5)	<0.001 ^a^
VLDL-c (mg/dL) Median (Q1, Q3)	27.7 ± 13.9 24.3 (18.0, 36.0)	25.9 ± 12.2 22.6 (17.0, 33.8)	33.0 ± 17.0 29.8 (21.0, 42.0)	0.002 ^a^
HDL-c (mg/dL)	39.9 ± 15.8	38.9 ± 13.4	42.7 ± 21.1	0.309
Hypercholesterolemia, n (%)	30 (16.3)	11 (8.0)	19 (40.4)	<0.001
Hypertriglyceridemia, n (%)	68 (37.0)	44 (32.1)	24 (51.1)	0.020
High LDL-c, n (%)	22 (14.7)	10 (8.8)	12 (32.4)	<0.001
Hypoalphalipoproteinemia, n (%)	108 (71.1)	81 (71.7)	27 (69.2)	0.771
AST (IU/L) Median (Q1,Q3)	65.7 ± 65.1 49.0 (29.0, 78.0)	78.0 ± 62.1 61.0 (40.0, 100.0)	33.7 ± 27.5 27.0 (23.9, 34.8)	<0.001 ^a^
ALT (IU/L) Median (Q1,Q3)	66.4 ± 58.5 45.0 (27.3, 78.0)	75.7 ± 70.9 57.5 (35.0, 91.8)	37.5 ± 31.4 27.0 (20.0, 43.5)	<0.001 ^a^
GGT (IU/L) Median (Q1,Q3)	69.9 ± 98.3 39.0 (24.0, 74.0)	77.5 ± 102.2 50.0 (27.0, 92.0)	48.7 ± 84.0 24.5 (20.0, 41.0)	<0.001 ^a^
*APOE* Allele Frequency ^b^				
*ε2*	14 (4.1)	12 (4.7)	2 (2.3)	0.323
*ε3*	297 (86.3)	224 (87.5)	73 (83.0)	0.284
*ε4*	33 (9.6)	20 (7.8)	13 (14.7)	0.056

Data are presented as mean ± SD unless indicated. Median, first quartile (Q1), and third quartile (Q3) are also presented. HCV, hepatitis C virus; BMI, body mass index; CHC, chronic hepatitis C; SC, spontaneous clearance; LDL-c, low-density lipoprotein-cholesterol; VLDL-c, very-low-density lipoprotein-cholesterol; HDL-c, high-density lipoprotein-cholesterol; ALT, alanine aminotransferase; AST, aspartate aminotransferase; and GGT, gamma-glutamyl transferase. The chi-square test was used to compare categorical variables, while the *t*-test and ^a^ Mann–Whitney U test were used for variables with normal and non-normal distributed data, respectively; ^b^ Frequency was obtained from 172 patients.

**Table 2:** Nutritional profile of chronic hepatitis C and spontaneous clearance patients according to *APOE* genotype group.

Variables	Chronic Hepatitis C			Spontaneous Clearance			RDA Values
	E2 (*n* = 10)	E3 (*n* = 100)	E4 (*n* = 18)	E2 (*n* = 2)	E3 (*n* = 30)	E4 (*n* = 12)	
Anthropometrics							
Weight (kg)	69.1 ± 5.5	70.5 ± 14.9	72.7 ± 18.3	75.9 ± 26.7	70.5 ± 9.6	80.0 ± 16.9	—
Body fat (%)	26.6 ± 10.9	31.4 ± 10.1	29.9 ± 10.6	30.1 ± 0.8	30.2 ± 9.0	31.8 ± 6.8	—
BMI (kg/m^2^)	26.1 ± 3.9	27.3 ± 5.2	27.8 ± 5.5	28.0 ± 4.6	27.0 ± 3.7	29.4 ± 4.9	—
Macronutrients							
Total energy (kcal)	2061 ± 446	2082 ± 696	1900 ± 510	2903 ± 4	2070 ± 470	2135 ± 414	—
Proteins (%)	15.7 ± 5.9	17.1 ± 4.0	16.1 ± 4.2	14.0 ± 1.4	16.0 ± 4.6	19.0 ± 4.9	15–20
Total fat (%)	24.5 ± 10.0	29.1 ± 8.2	26.3 ± 7.9	24.0 ± 7.1	29.2 ± 9.3	29.9 ± 3.9	25–30
SFA (%)	5.9 ± 4.4	7.8 ± 3.3	6.8 ± 2.7	6.5 ± 3.5	8.2 ± 3.6	7.1 ± 3.2	<7
MUFA (%)	8.1 ± 5.1	9.9 ± 4.0	8.9 ± 4.0	8.0 ± 1.4	9.6 ± 4.6	11.2 ± 6.9	10–15
PUFA (%)	3.8 ± 1.5	4.8 ± 2.2	4.5 ± 1.7	4.0 ± 0.0	4.6 ± 2.1	5.6 ± 3.2	7–10
Cholesterol (mg/dL)	217.8 ± 166.3	311.4 ± 251.6	239.5 ± 155.0	125.0 ± 0.0	300.1 ± 248.9	334.5 ± 207.3	<200
Carbohydrates (%)	61.4 ± 12.9	55.9 ± 10.0	59.8 ± 9.9	64.0 ± 7.1	57.1 ± 12.4	52.6 ± 3.8	50–55
Fiber (g/day)	18.9 ± 8.3	25.3 ± 12.7	19.3 ± 8.3	28.3 ± 0.0	24.2 ± 12.7	25.2 ± 1.5	25–38
Sugar (g/day)	29.0 ± 20.3	36.2 ± 26.4	48.8 ± 39.0	40.1 ± 0.0	48.6 ± 48.6	32.8 ± 24.9	<30
Micronutrients ^a^							
Vitamin A (μg/day)	1407.9 ± 2288.7	1045.1 ± 1138.9	1493.6 ± 1689.7	831 ± 0.0	950.1 ± 777.8	1184.1 ± 535.2	900
Vitamin E (mg/day)	1.7 ± 1.0	2.6 ± 2.0	2.0 ± 1.6	3.8 ± 0.0	2.4 ± 2.2	2.7 ± 1.9	15
Folates (μg/day of DFE)	98.3 ± 101.4	189.2 ± 148.1	157.5 ± 80.2	90.0 ± 0.0	173.6 ± 147.9	366.1 ± 360.6	300–600
Thiamin (mg/day)	1.2 ± 0.5	1.3 ± 0.7	1.2 ± 0.6	2.0 ± 0.0	1.3 ± 0.6	1.7 ± 1.3	1.1–1.2
Pyridoxine (mg/day)	1.0 ± 0.3	1.3 ± 0.8	1.5 ± 0.9	0.6 ± 0.0	1.2 ± 0.9	1.8 ± 1.4	1.7
Cobalamin (μg/day)	3.1 ± 3.3	3.5 ± 2.5	2.7 ± 2.1	4.4 ± 0.0	2.5 ± 1.9	4.8 ± 3.3	2.4
Iron (mg/day)	14.3 ± 6.3	16.2 ± 7.2	13.4 ± 4.4	27.3 ± 0.0	14.2 ± 5.6	20.5 ± 12.6	8–18
Selenium (μg/day)	30.4 ± 27.5	42.6 ± 37.8	26.6 ± 19.7	47.0 ± 0.0	42.7 ± 36.4	59.1 ± 73.5	55

BMI, body mass index; SFA, saturated fatty acids; MUFA, monounsaturated fatty acids; PUFA, polyunsaturated fatty acids; DFE, dietary folate equivalents; and RDA, Recommended Dietary Allowance. A total of 172 study patients had *APOE* genotyping data. ^a^ Only vitamins and minerals with a potential effect on liver function are listed. Differences between variables according to the *APOE* genotype group in CHC and SC patients were evaluated with the Kruskal–Wallis test. No significant differences were found.

**Table 3:** Nutritional and biochemical characteristics of CHC patients according to viral load.

		Chronic Hepatitis C Patients		
Variables	Reference Values	Low Viral Load (n = 36)	High Viral Load (n = 101)	*p*-Value
Anthropometrics				
Body fat (%)	—	30.7 ± 10.3	30.4 ± 10.3	0.882
BMI (kg/m^2^)	—	27.7 ± 5.3	26.9 ± 5.5	0.458
Macro- and Micronutrients	Ref [46]			
Total energy (kcal) Median (Q1, Q3)	—	1926 ± 483 1852 (1562, 2322)	2126 ± 713 1970 (1685, 2555)	0.233 ^a^
Proteins (%)	15–20	17.7 ± 4.5	16.5 ± 4.0	0.138
Total fat (%)	25–30	30.9 ± 8.2	27.5 ± 8.0	0.030
SFA (%)	<7	8.0 ± 3.3	7.5 ± 3.3	0.449
MUFA (%)	10–15	10.4 ± 4.1	9.4 ± 4.0	0.216
PUFA (%)	7–10	5.4 ± 2.7	4.4 ± 1.9	0.030
Cholesterol (mg/dL) Median (Q1, Q3)	<200	316.0 ± 231.8 229.5 (147.5, 431.0)	278.5 ± 236.5 199 (107.0, 415.8)	0.239 ^a^
Carbohydrates (%)	50–55	54.1 ± 9.6	57.9 ± 10.2	0.052
Fiber (g/day)	25–38	19.8 ± 9.5	26.3 ± 13.8	0.016
Sugar (g/day)	<30	33.3 ± 28.9	39.0 ± 26.6	0.314
Vitamin A (μg/day)	900	1050.0 ± 1268.1	1099.4 ± 1314.0	0.844
Vitamin E (mg/day)	15	2.3 ± 1.9	2.4 ± 1.9	0.742
Laboratory Data	Ref [33]			
Glucose (mg/dL) Median (Q1, Q3)	≤100	103.5 ± 34.8 96.5 (84.3, 109.8)	114.4 ± 58.7 97.0 (87.0, 116.5)	0.334 ^a^
ALT (IU/L) Median (Q1, Q3)	≤42	53.3 ± 34.7 42.5 (29.5, 70.3)	83.8 ± 78.6 61.5 (37.0, 103.8)	0.011 ^a^
AST (IU/L) Median (Q1, Q3)	≤54	62.7 ± 40.1 52.5 (35.5, 77.5)	83.6 ± 67.7 65.0 (41.0, 107.0)	0.083 ^a^
GGT (IU/L) Median (Q1, Q3)	≤30	44.0 ± 27.4 33.5 (26.3, 64.5)	88.8 ± 115.1 54.0 (28.0, 110.0)	0.013 ^a^
Platelets (×10^3^/μL)	150–450	161.7 ± 116.4	150.0 ± 71.0	0.590
Albumin (g/dL)	3.4–5.4	3.4 ± 0.6	3.6 ± 0.6	0.047
T. bilirubin (mg/dL) Median (Q1, Q3)	0.1–1.2	1.2 ± 0.9 1.0 (0.6, 1.5)	1.3 ± 1.6 0.9 (0.6, 1.4)	0.935 ^a^

Data are presented as mean ± SD unless otherwise indicated. Median, first quartile (Q1), and third quartile (Q3) are also presented. CHC, chronic hepatitis C; BMI, body mass index; SFA, saturated fatty acids; MUFA, monounsaturated fatty acids; PUFA, polyunsaturated fatty acids; ALT, alanine aminotransferase; AST, aspartate aminotransferase; GGT, gamma-glutamyl transferase; and T. bilirubin, total bilirubin. Statistical analyses were performed using the *t*-test or ^a^ Mann–Whitney U test.

**Table 4:** Logistic regression analysis of dietary variables associated with high viral load in CHC patients.

Dietary Variable	Univariate			Multivariate		
	OR	95 % CI	*p*-Value	OR	95 % CI	*p*-Value
Total fat (%)	0.949	0.904–0.996	0.032			
PUFA (%)	0.805	0.668–0.969	0.022	0.804	0.656–0.986	0.036
Carbohydrates (%)	1.040	0.999–1.082	0.055			
Fiber (g/day)	1.050	1.008–1.093	0.019	1.049	1.007–1.092	0.021

OR, odds ratio; CI, confidence interval; PUFA, polyunsaturated fatty acids. Variables with *p* < 0.2 in the univariate analysis were included for multivariate analysis. Hosmer–Lemeshow test: chi-square = 3.9, *p* = 0.866; Nagelkerke R square = 0.135.

**Table 5:** Receiver-operating characteristic analyses of dietary variables associated with low or high viral load.

HCV Viral Load	Variable	Cut-Off	AUC	*p*-Value	Sensitivity, %	Specificity, %
Low	PUFA (%)	≥4.9	0.624	0.032	61.8	56.6
High	Fiber (g/day)	≥21.5	0.633	0.026	61.4	69.7

HCV, hepatitis C virus; PUFA, polyunsaturated fatty acids; AUC, area under the curve.

**Table 6:** Frequency of CHC patients with HCV-related variables relative to adherence to dietary patterns.

Dietary Patterns		HCV-Related Variables			
	Adherence Tertile	PUFA ≥4.9%	Fiber ≥21.5 g/day	Low Viral Load	High Viral Load
Meat and soft drinks, DP1	T1	19 (54.4)	20 (57.1)	13 (36.1)	23 (63.9)
	T2	15 (42.9)	12 (46.7)	5 (13.9)	31 (86.1)
	T3	14 (40.0)	16 (50.0)	10 (27.8)	26 (72.2)
Processed animal and fried foods, DP2	T1	16 (38.1)	19 (45.2)	8 (19.0)	34 (81.0)
	T2	15 (45.5)	16 (57.1)	11 (32.4)	23 (67.6)
	T3	17 (56.7)	15 (55.6)	9 (28.1)	23 (71.9)
Mexican-healthy DP3	T1	17 (50.0)	20 (62.5)	10 (28.6)	25 (71.4)
	T2	15 (41.7)	14 (42.4)	11 (29.7)	26 (70.3)
	T3	16 (45.7)	16 (50.0)	7 (19.4)	29 (80.6)
Fish-rich DP4	T1	11 (29.7)	24 (68.6)	6 (16.2)	31 (83.8)
	T2	16 (42.1)	13 (39.4)	10 (25.0)	30 (75.0)
	T3	21 (70.0) ^a^	13 (44.8) ^b^	12 (38.7) ^c^	19 (61.3)

Values are expressed as numbers and percentages; HCV, hepatitis C virus; and PUFA, polyunsaturated fatty acids. Comparisons were performed with chi-square test. ^a^ Fish-rich T3 vs. T1, *p* = 0.004; ^b^ fish-rich T3 vs. T1, *p* = 0.038; ^c^ fish-rich T3 vs. T1, *p* = 0.036.

## Data Availability

The data presented in this study are available on request from the corresponding author. The data are not publicly available due to policies of patients’ data privacy.

## Supplementary Materials

The following are available online at https://www.mdpi.com/article/10.3390/nu13103337/s1, Table S1: Factor loading matrix of main dietary patterns identified in anti-HCV-positive patients.
