# Supplementary material for: Adherence to a Fish-Rich Dietary Pattern Is Associated with Chronic Hepatitis C Patients Showing Low Viral Load: Implications for Nutritional Management
Source: Nutrients. 2021 Sep 23;13(10):3337. doi: 10.3390/nu13103337 (PMC8541240; doi:10.3390/nu13103337)
Supplement: Supplementary file 1 [file nutrients-13-03337-s001.zip › nutrients-1387136-supplementary.pdf]

Table S1. Factor loading matrix of main dietary patterns identified in anti-HCV positive patients.

Food groups	Dietary patterns			
	Meat and soft drinks DP1	Processed animal and fried foods DP2	Mexican-healthy DP3	Fish-rich DP4
Tortilla	-0.009	0.012	-0.080	0.113
Whole grains	-0.029	-0.002	0.122	-0.042
Legumes	-0.008	0.016	0.064	0.026
Fruits	-0.077	-0.164	**0.770**	0.019
Vegetables	-0.013	0.022	**0.502**	0.100
Red meat	**0.665**	0.188	0.036	0.065
Chicken	0.067	0.057	0.214	0.286
Pork	**0.749**	0.238	-0.101	0.037
Fish	0.071	0.041	0.110	**0.896**
Seafood	0.081	0.000	-0.121	0.255
Milk and dairy	-0.130	-0.111	-0.010	-0.017
Vegetable oils	0.025	-0.079	0.206	0.186
Avocado	0.011	0.119	-0.150	0.141
Processed meat	0.165	**0.693**	0.070	0.124
Bacon	**0.308**	**0.456**	-0.256	-0.077
Cream, mayonnaise, dressing	0.175	**0.504**	-0.033	0.019
Butter, margarine	0.091	0.181	-0.056	-0.158
Fried foods	0.105	**0.376**	-0.157	-0.027
Nuts	0.005	-0.024	0.090	0.007
Soft drinks	**0.335**	0.087	-0.078	0.034
Coffee	0.260	0.064	0.000	0.075
Baked goods	0.002	0.084	0.014	0.031
Sugar, sweets	-0.103	0.026	-0.097	0.010

DP, dietary pattern. In bold are those food groups representative of each DP based on a factor loading cutoff value score of ≥ 0.30.
